# Supplementary material for: Growth Quality and Development of Olive Plants Cultured In-Vitro under Different Illumination Regimes
Source: Plants (Basel). 2021 Oct 18;10(10):2214. doi: 10.3390/plants10102214 (PMC8541116; doi:10.3390/plants10102214)
Supplement: Supplementary file 1 [file plants-10-02214-s001.zip › 2021_10_04 Supplementary FIGURES.pptx]

## Slide 1
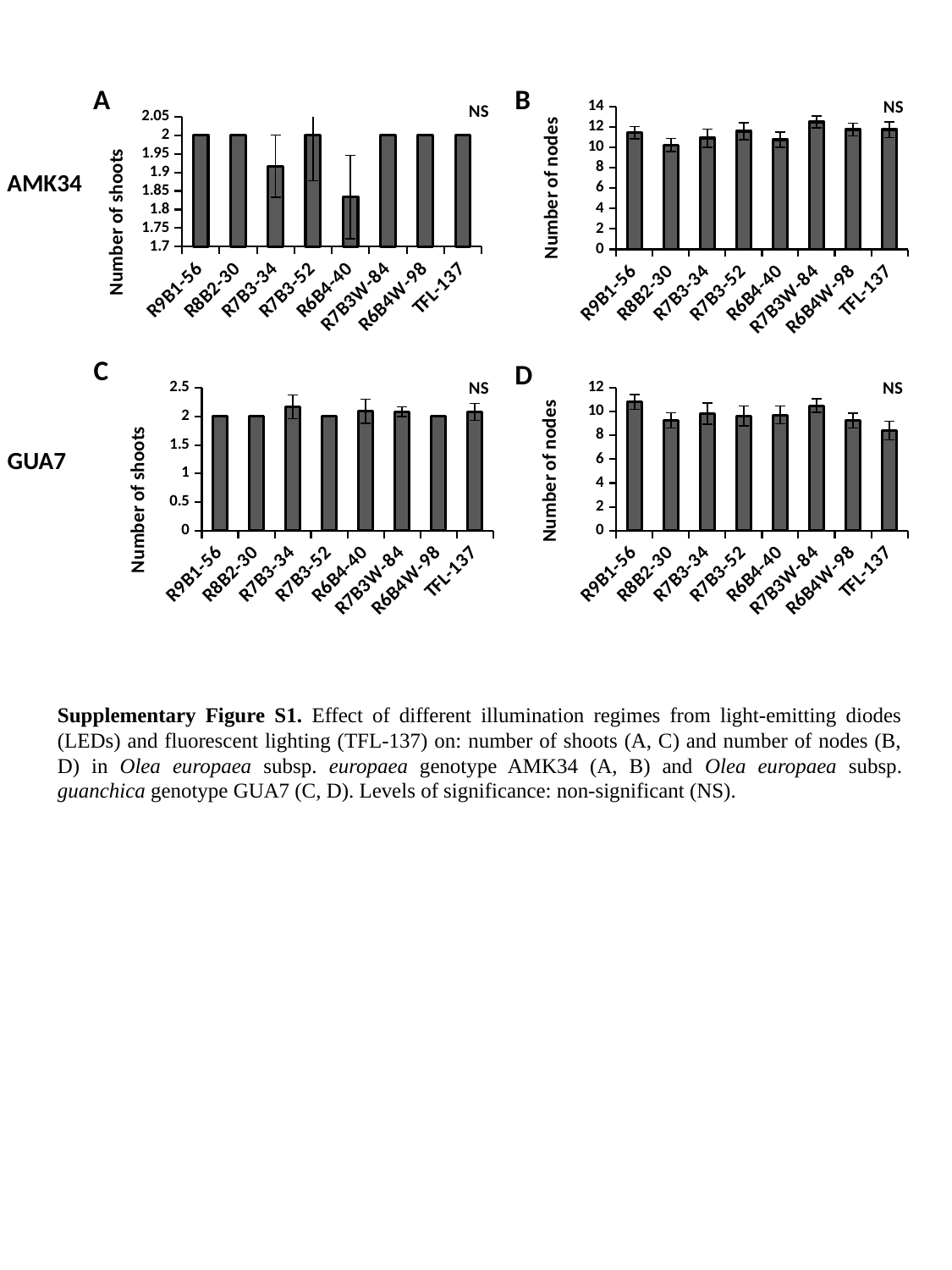

B
A
### Chart
| Category | Nº Nudos |
|---|---|
| R9B1-56 | 11.454545454545455 |
| R8B2-30 | 10.25 |
| R7B3-34 | 10.916666666666666 |
| R7B3-52 | 11.583333333333334 |
| R6B4-40 | 10.75 |
| R7B3W-84 | 12.5 |
| R6B4W-98 | 11.75 |
| TFL-137 | 11.75 |
### Chart
| Category | Long tallo |
|---|---|
| R9B1-56 | 2.0 |
| R8B2-30 | 2.0 |
| R7B3-34 | 1.9166666666666667 |
| R7B3-52 | 2.0 |
| R6B4-40 | 1.8333333333333333 |
| R7B3W-84 | 2.0 |
| R6B4W-98 | 2.0 |
| TFL-137 | 2.0 |AMK34
C
D
### Chart
| Category | Nº brotes |
|---|---|
| R9B1-56 | 2.0 |
| R8B2-30 | 2.0 |
| R7B3-34 | 2.1666666666666665 |
| R7B3-52 | 2.0 |
| R6B4-40 | 2.090909090909091 |
| R7B3W-84 | 2.0833333333333335 |
| R6B4W-98 | 2.0 |
| TFL-137 | 2.0833333333333335 |
### Chart
| Category | Nº Nudos |
|---|---|
| R9B1-56 | 10.818181818181818 |
| R8B2-30 | 9.272727272727273 |
| R7B3-34 | 9.833333333333334 |
| R7B3-52 | 9.636363636363637 |
| R6B4-40 | 9.727272727272727 |
| R7B3W-84 | 10.5 |
| R6B4W-98 | 9.25 |
| TFL-137 | 8.416666666666666 |GUA7
Supplementary Figure S1. Effect of different illumination regimes from light-emitting diodes (LEDs) and fluorescent lighting (TFL-137) on: number of shoots (A, C) and number of nodes (B, D) in Olea europaea subsp. europaea genotype AMK34 (A, B) and Olea europaea subsp. guanchica genotype GUA7 (C, D). Levels of significance: non-significant (NS).

## Slide 2
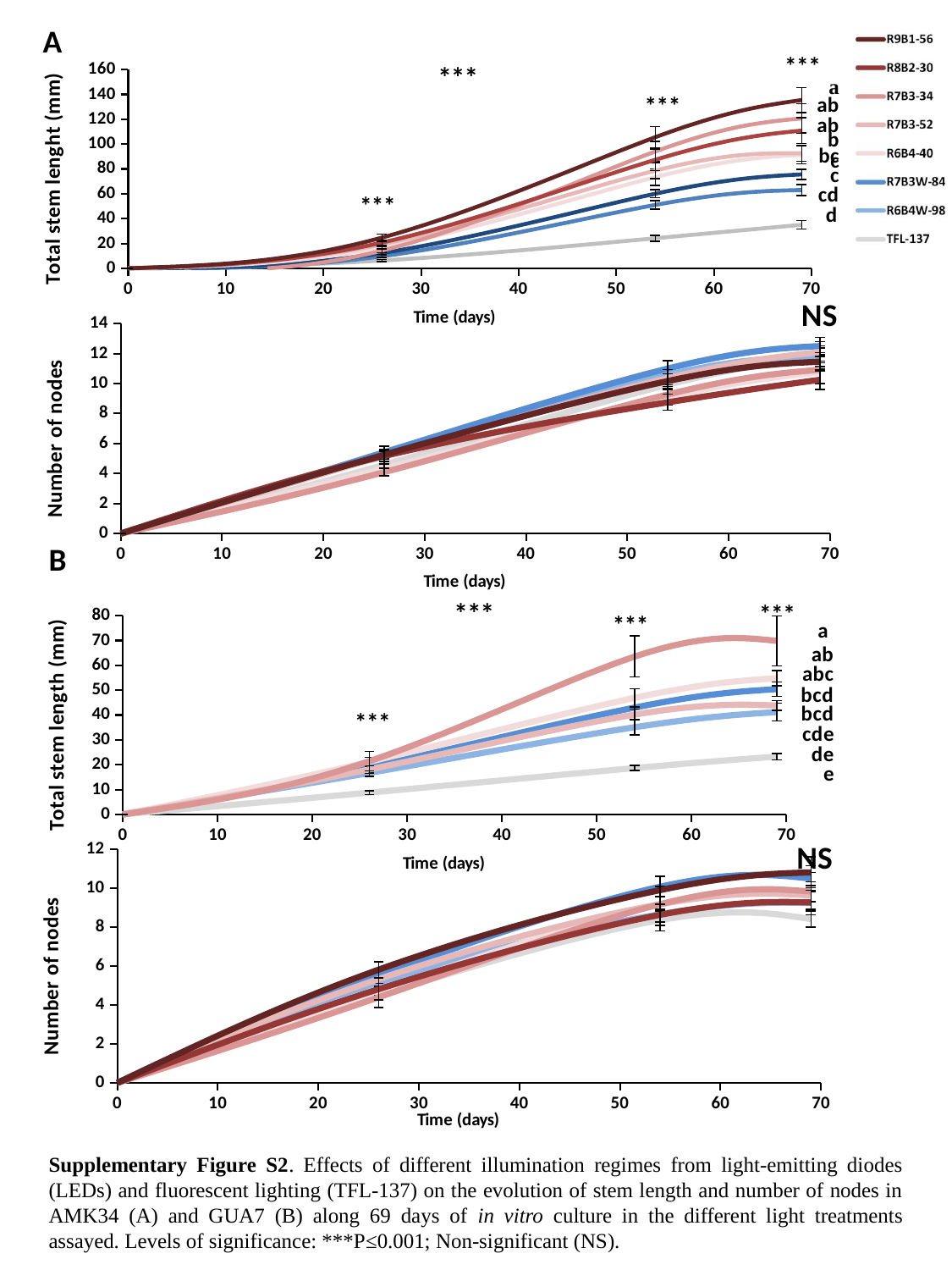

A
### Chart
| Category | R9B1-56 | R8B2-30 | R7B3-34 | R7B3-52 | R6B4-40 | RW7B3-84 | RW6B4-98 | TFL-137 |
|---|---|---|---|---|---|---|---|---|
### Chart
| Category | R9B1-56 | R8B2-30 | R7B3-34 | R7B3-52 | R6B4-40 | RW7B3-84 | RW6B4-98 | TFL-137 |
|---|---|---|---|---|---|---|---|---|B
### Chart
| Category | R9B1-56 | R8B2-30 | R7B3-34 | R7B3-52 | R6B4-40 | RW7B3-84 | RW6B4-98 | TFL-137 |
|---|---|---|---|---|---|---|---|---|
### Chart
| Category | R9B1-56 | R8B2-30 | R7B3-34 | R7B3-52 | R6B4-40 | RW7B3-84 | RW6B4-98 | TFL-137 |
|---|---|---|---|---|---|---|---|---|Supplementary Figure S2. Effects of different illumination regimes from light-emitting diodes (LEDs) and fluorescent lighting (TFL-137) on the evolution of stem length and number of nodes in AMK34 (A) and GUA7 (B) along 69 days of in vitro culture in the different light treatments assayed. Levels of significance: ***P≤0.001; Non-significant (NS).

## Slide 3
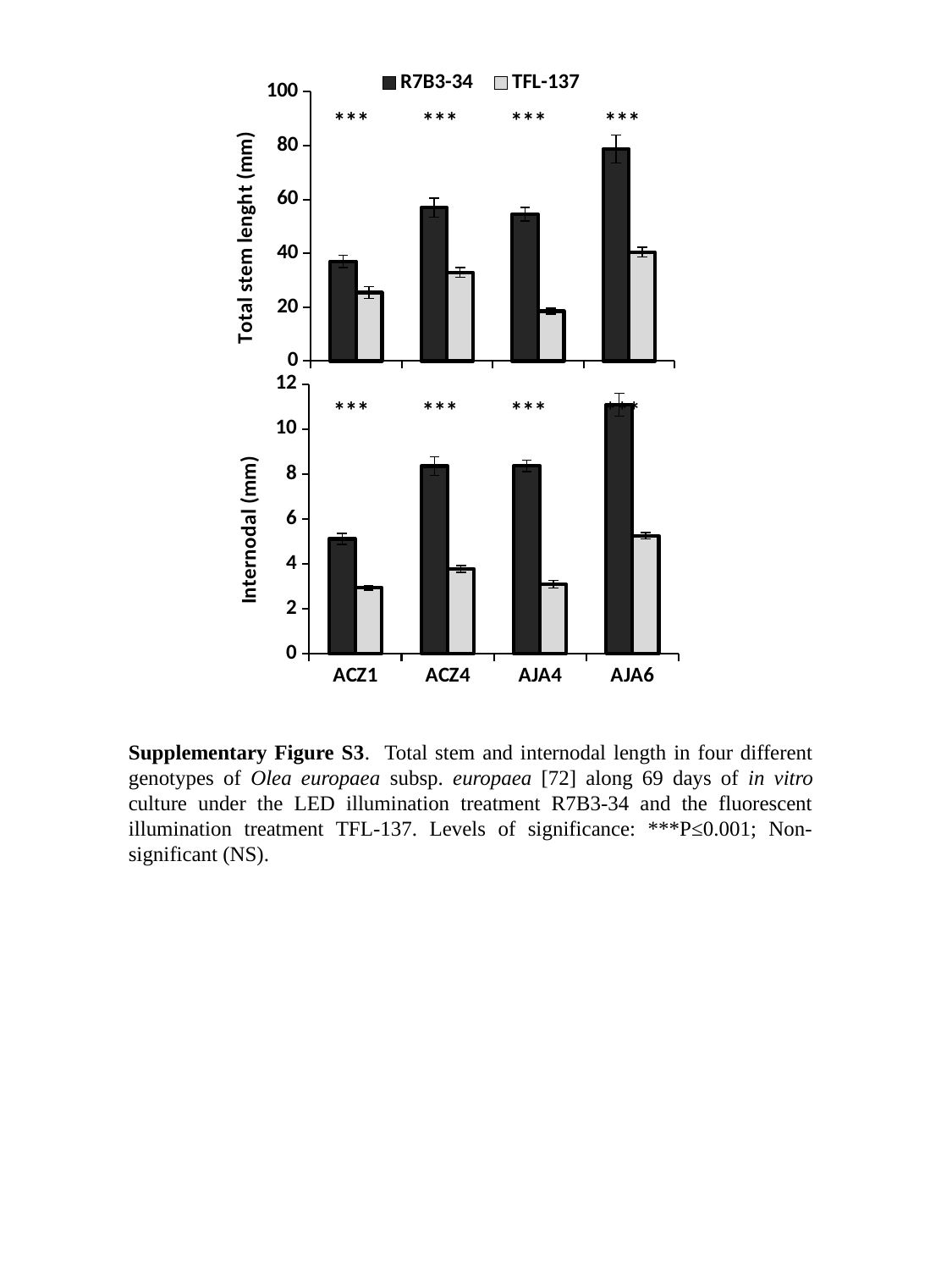

### Chart
| Category | R7B3-34 | TFL-137 |
|---|---|---|
| ACZ1 | 37.0 | 25.4 |
| ACZ4 | 56.92307692307692 | 32.88 |
| AJA4 | 54.53846153846154 | 18.53846153846154 |
| AJA6 | 78.70370370370371 | 40.48148148148148 |
### Chart
| Category | R7B3-34 | TFL-137 |
|---|---|---|
| ACZ1 | 5.105672438672439 | 2.9240115995115996 |
| ACZ4 | 8.350463425463426 | 3.7676987456987456 |
| AJA4 | 8.354817404817407 | 3.0792277167277153 |
| AJA6 | 11.082275132275132 | 5.238470685692908 |Supplementary Figure S3. Total stem and internodal length in four different genotypes of Olea europaea subsp. europaea [72] along 69 days of in vitro culture under the LED illumination treatment R7B3-34 and the fluorescent illumination treatment TFL-137. Levels of significance: ***P≤0.001; Non-significant (NS).

## Slide 4
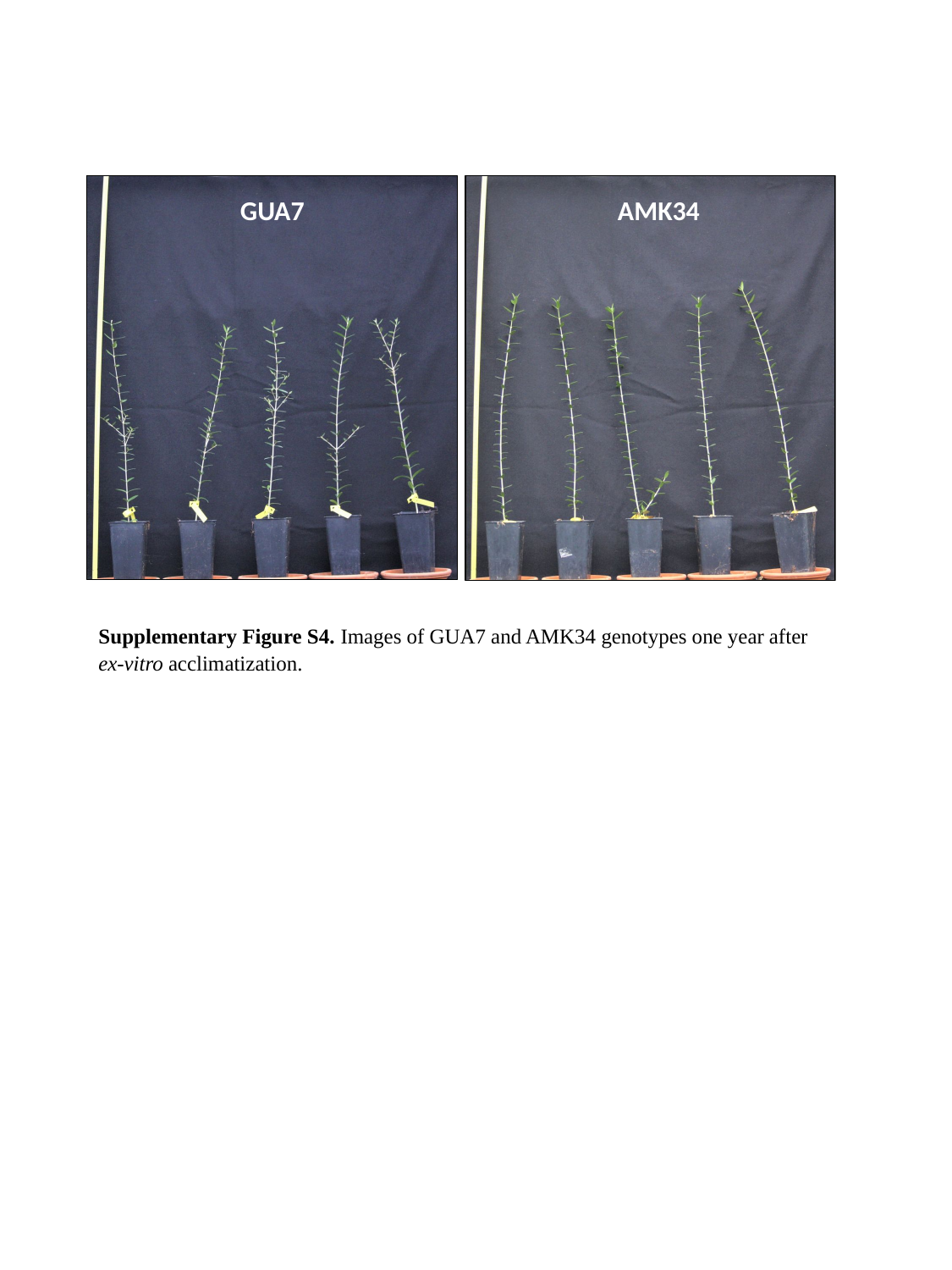

Supplementary Figure S4. Images of GUA7 and AMK34 genotypes one year after ex-vitro acclimatization.

## Slide 5
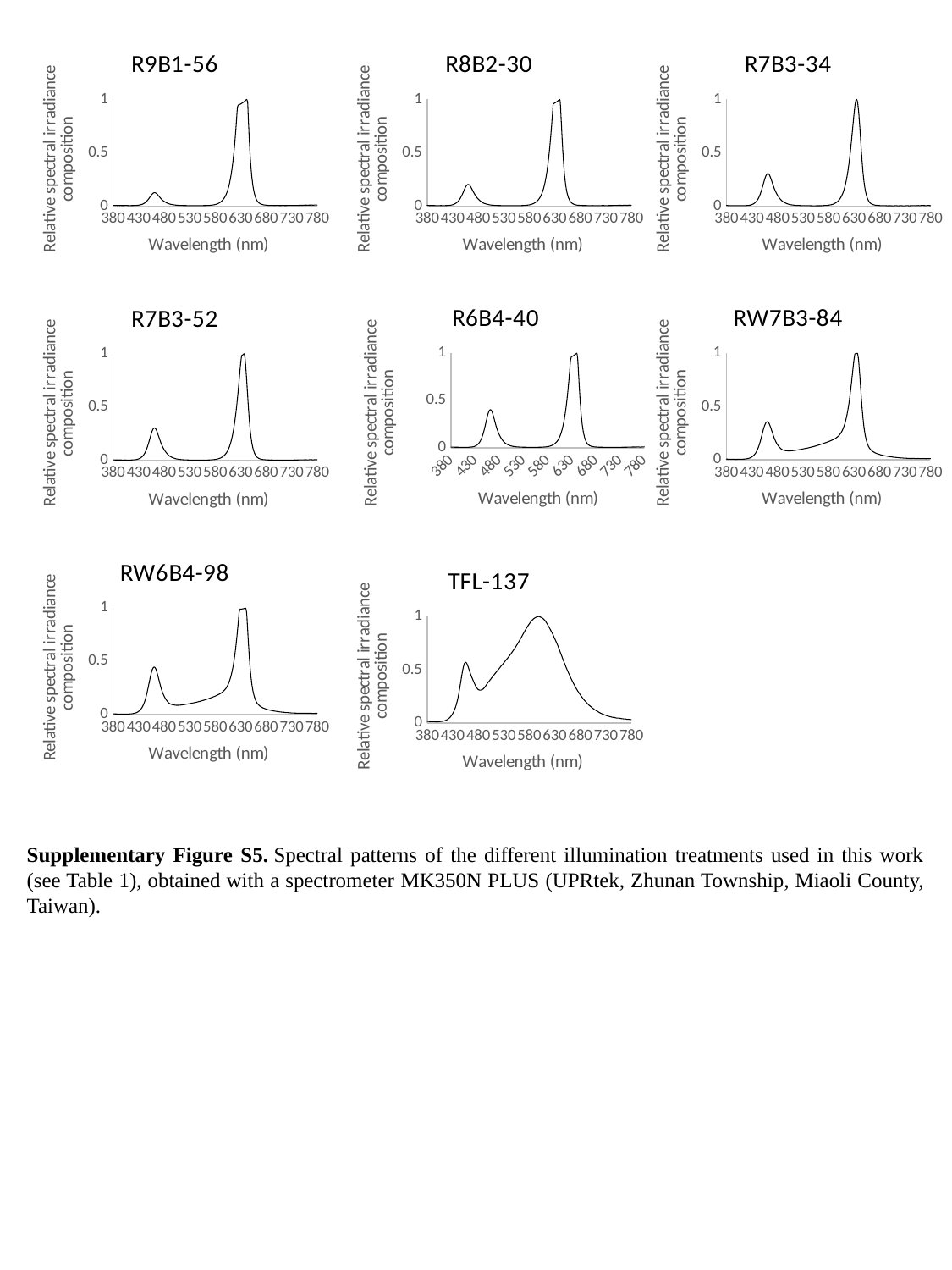

### Chart: R9B1-56
| Category | |
|---|---|
### Chart: R8B2-30
| Category | |
|---|---|
### Chart: R7B3-34
| Category | |
|---|---|
### Chart: R6B4-40
| Category | |
|---|---|
### Chart: RW7B3-84
| Category | |
|---|---|
### Chart: R7B3-52
| Category | |
|---|---|
### Chart: RW6B4-98
| Category | |
|---|---|
### Chart: TFL-137
| Category | |
|---|---|Supplementary Figure S5. Spectral patterns of the different illumination treatments used in this work (see Table 1), obtained with a spectrometer MK350N PLUS (UPRtek, Zhunan Township, Miaoli County, Taiwan).
